# Murine typhus: An unlikely cause of cat scratch fever with ascending rash

**DOI:** 10.1016/j.jdcr.2024.12.023

**Published:** 2025-01-28

**Authors:** Victoria Jiminez, Anna Catinis, Ida Orengo, Janna Nunez-Gussman

**Affiliations:** a Heersink School of Medicine, University of Alabama at Birmingham, Birmingham, Alabama; b Department of Dermatology, Baylor College of Medicine, Houston, Texas; c Christus Southeast Texas Hospital-St. Elizabeth, Beaumont, Texas

**Keywords:** ascending rash, cat scratch fever, flea-borne illness, murine typhus, Rickettsia typhi

## Introduction

Murine typhus is a flea-borne febrile illness caused by the bacteria Rickettsia typhi. It is often misdiagnosed due to its nonspecific symptoms, including fever, malaise, and headache.^1^ Common vectors of murine typhus include rodents like rats, opossums, and cats.^2^ While R. typhi infections are reported worldwide, they are rare in the United States, primarily occurring in southern Texas and California.^2^ Here, we present the case of a female patient with a fever of unknown origin, localized rash, and visual floaters, who was found to have murine typhus after a cat scratch in southeast Texas.

## Clinical presentation

A 55-year-old female presented to the emergency department with a 5-day history of intractable fever up to 39 degrees Celsius, headache, myalgias, night sweats, and nausea. She initially presented to the primary care physician 2 days after symptom onset, where Entero/Rhinovirus was positive, despite having no upper respiratory symptoms. Aspartate aminotransferase and alanine aminotransferase were elevated, which prompted the patient’s presentation to the Emergency Department. Initial exam was nonspecific, and vital signs were remarkable for fever and tachycardia. Past medical history was noncontributory.

**Fig 1:**
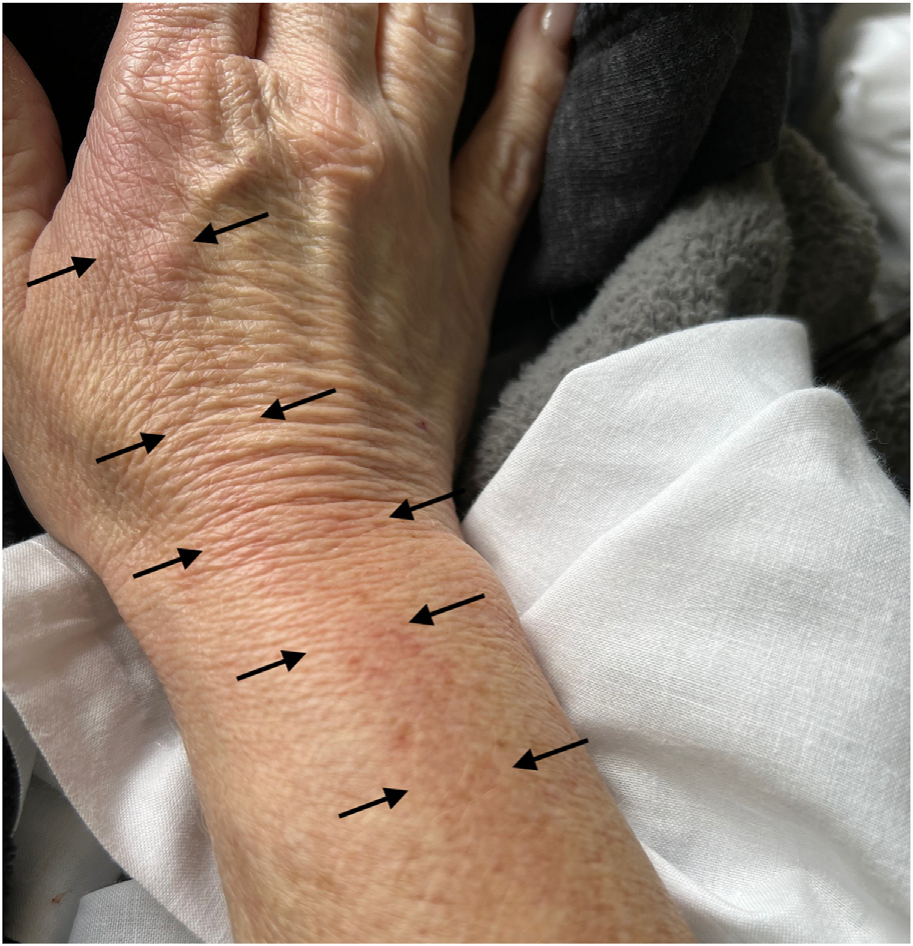
Erythema (highlighted by the *black arrows* on either side) of the right index finger with proximal spread to the dorsal hand and wrist.

Laboratory analysis revealed lactic acidosis, thrombocytopenia, hypokalemia, and hypoalbuminemia. Urinalysis showed occult blood, red blood cells, and granular casts. Aspartate aminotransferase, alanine aminotransferase, alkaline phosphatase levels, and lipase were elevated at 142, 172, 290, and 114 (U/L), respectively. Computed tomography of the abdomen revealed mild splenomegaly. Ten days prior to symptom onset, the patient revealed she had been scratched on the right index finger by a kitten she was fostering. She then developed a small erythematous papule on the finger and noticed erythema that spread proximally to involve the right dorsal hand and wrist (Fig 1). Infectious disease was consulted, and the patient was started on empiric vancomycin and piperacillin-tazobactam.

Labs were notable for an acute hemoglobin drop from 14.7 to 9.9 (g/dL) on day 2 of admission. Lactate dehydrogenase, procalcitonin, D-dimer, coagulation factors, C-reactive protein, and ferritin were elevated. On day 3, the patient remained febrile with new leukocytosis from 5.6 to 12.4 (cells/uL), prompting an antibiotic change to azithromycin and doxycycline for coverage of suspected atypical bacterial infection.

On day 4, the patient reported new photophobia and visual floaters, raising concerns for retinal involvement of a systemic infection. Throughout the admission, an extensive workup, including peripheral smear, flow cytometry, immunoglobulins, serum protein electrophoresis, antinuclear antibody, cytomegalovirus, human immunodeficiency virus, hepatitis panels, and blood cultures, was unrevealing. On day 5, labs began to improve with normalization of the leukocytosis, decreasing transaminases, and the patient was discharged and completed the 10-day course of doxycycline.

At outpatient follow-up, serologies obtained during the patient’s admission resulted and were notably positive for Rickettsia rickettsii titers, while titers for Toxoplasma and *Bartonella henselae* were negative. The patient's lack of tick or rodent exposure made Rocky Mountain Spotted Fever unlikely. The ophthalmologic symptoms prompted further testing for the typhus fever group of rickettsial agents.

Two months after the patient’s initial presentation, elevated titers for R. typhi IgG (>1:256) and IgM (>1:256) resulted, with IgG and IgM of R. rickettsia titers remaining negative, confirming a diagnosis of murine typhus. Ophthalmologic follow-up examination revealed disseminated chorioretinitis. Repeat computed tomography after 1 month showed improved lymphadenopathy. At 5-month follow-up, the patient had no residual symptoms.

## Discussion

Murine typhus is an acute febrile illness characterized by symptoms such as fever, headache, myalgias, gastrointestinal symptoms, and a truncal rash, leading to hospitalization in 10% of patients.^2^^,^^3^ The typhus fever group of rickettsial agents includes *R. typhi*, *R.prowazekii*, and *O. tsustugamushi*. Murine typhus is caused by *R. typhi* and is also known as flea-borne typhus or endemic typhus.^1^ Transmission typically involves rodent vectors, but many patients never recall a specific exposure history.^2^^,^^4^

Delayed diagnosis and treatment of murine typhus can result in a prolonged fever lasting weeks and may lead to end-organ damage or fatality if left untreated.^1^ Severe cases often present with early laboratory abnormalities, as observed in this patient.^5^ Murine typhus has been associated with secondary hemophagocytic syndrome, characterized by cytopenias, hyperferritinemia, and splenomegaly, which were also present in this case.^6^ As with other rickettsial infections, doxycycline remains the mainstay of treatment.^1^

Our patient presented with the symptomatic triad of headache, fever, and rash, a combination seen in only one-third of murine typhus cases, along with hallmark lab abnormalities.^1^ However, the ascending hand rash is an atypical presentation compared to the classically reported maculopapular rash on the trunk and extremities.^5^ The clinical presentation in this case shares features with other infectious diseases, such as the visual floaters and chorioretinitis seen in toxoplasmosis, and localized rash with lymphadenopathy characteristic of cat scratch disease caused by *Bartonella henselae*.^7^^,^^8^ Additionally, the serologies may reflect potential cross-reactivity among different Rickettsial species titers.^9^

The ophthalmologic manifestations of R. typhi seen in this case are rarely seen, but existing reports aided in reaching the diagnosis.^3^ Early fundoscopic examination and thorough skin examination can help refine the differential diagnosis while awaiting serologic results.^10^ Given the low risk associated with doxycycline, its administration is justified prior to confirmatory diagnosis of murine typhus, as early treatment can prevent serious complications, as evidenced by this case.

Diagnosis of murine typhus in this case may have been complicated and delayed by a positive viral test, concerns over potential acetaminophen-induced transaminitis, and recent travel history. While doxycycline effectively prevented end-organ damage, the patient experienced prolonged effects of *R. typhi* infection, including slowly resolving anemia and lymphadenopathy. This case represents a unique presentation of murine typhus, characterized by an identifiable mammal exposure, an atypical rash, systemic infection, and disseminated chorioretinitis.

## Conclusion

It is critical to consider murine typhus when presented with a patient with cat, rodent, or flea exposure with systemic symptoms, rash, and visual floaters, especially in endemic geographical areas. For patients with undifferentiated clinical manifestations or localized rash, life-saving doxycycline can be administered empirically to prevent severe and potentially permanent sequelae.

## Conflicts of interest

None disclosed.
